# Sheep and Goats Respond Differently to Feeding Strategies Directed to Improve the Fatty Acid Profile of Milk Fat

**DOI:** 10.3390/ani10081290

**Published:** 2020-07-28

**Authors:** Anna Nudda, Antonello Cannas, Fabio Correddu, Alberto Stanislao Atzori, Mondina Francesca Lunesu, Gianni Battacone, Giuseppe Pulina

**Affiliations:** Dipartimento di Agraria, Sezione di Scienze Zootecniche, University of Sassari, Viale Italia, 3907100 Sassari, Italy; anudda@uniss.it (A.N.); cannas@uniss.it (A.C.); asatzori@uniss.it (A.S.A.); mflunesu@uniss.it (M.F.L.); battacon@uniss.it (G.B.); gpulina@uniss.it (G.P.)

**Keywords:** sheep milk, goat milk, fatty acid, grass, fish oil, secondary metabolites

## Abstract

**Simple Summary:**

Sheep and goat milk, as well as dairy products, are considered good sources of high-quality nutrients, particularly proteins and fats. Many positive effects on human health have been attributed to the consumption of dairy containing specific fatty acids, including some compounds originating from the polyunsaturated FA (PUFA) biohydrogenation operated by rumen microbes. In this bibliographic review, several nutritional strategies able to improve the milk fatty acids (FA) profile, in terms of an increase in the concentration of fatty acids considered beneficial to human health, are presented and discussed, with special attention to the differences between the two species.

**Abstract:**

This bibliographic review presents and discusses the nutritional strategies able to increase the concentration of beneficial fatty acids (FA) in sheep and goat milk, and dairy products, with a particular focus on the polyunsaturated FA (PUFA), and highlights differences between the two species. In fact, by adopting appropriate feeding strategies, it is possible to markedly vary the concentration of fat in milk and improve its FA composition. These strategies are based mostly on the utilization of herbage rich in PUFA, or on the inclusion of vegetable, marine, or essential oils in the diet of lactating animals. Sheep respond more effectively than goats to the utilization of fresh herbage and to nutritional approaches that improve the milk concentration of c9,t11-conjugated linoleic acid (c9,t11-CLA) and α-linolenic acid. Dietary polyphenols can influence milk FA profile, reducing or inhibiting the activity and growth of some strains of rumen microbes involved in the biohydrogenation of PUFA. Although the effectiveness of plant secondary compounds in improving milk FA composition is still controversial, an overall positive effect has been observed on the concentration of PUFA and RA, without marked differences between sheep and goats. On the other hand, the positive effect of dietary polyphenols on the oxidative stability of milk fat appears to be more consistent.

## 1. Introduction

During the last 50 years, world consumption of dairy products from goats and sheep has more than doubled and, as predicted by Pulina et al. [1], this trend will continue to rise until 2030 by 53% and 26% for goats and sheep, respectively.

Total goat and sheep milk production is estimated at 18.7 and 10.6 million tons, respectively [2]. In terms of percentage, this represents only 1.9% and 1.3% (for goat and sheep milk, respectively) of global milk production [1].

Regarding goat milk, Asia is the largest producer (57%; 10.6 million tons), followed by Africa (24%; 4.6 million tons), Europe (15%; 2.7 million tons), and the Americas (4%; 780 thousand tons) [2]. Likewise, sheep milk is mostly produced in Asia (46%; 4.9 million tons), followed by Europe (30%; 3.2 million tons), Africa (23%; 2.4 million tons), and the Americas (1%; 91 thousand tons) [2]. Goat dairy products are represented mainly by yogurt and cheese, followed by fermented milk and curd [3]. The greatest amount of goat cheese came from Eastern Africa, followed by Western Europe (110,750 and 87,407 tons, respectively) [3]. With respect to goat milk, almost all of the sheep milk is directed toward cheese-making; Italy is the leader in sheep cheese exports, with 36% of the market share [1].

Sheep milk and dairy products are considered good sources of high-quality nutrients, particularly proteins and fats. The research on milk fat is still oriented to the improvement of its nutritional value, with particular attention paid to increasing the content of fatty acids (FA) considered beneficial to human health, such as branched-chain fatty acids (BCFA), ruminant trans-fatty acids (TFA), especially rumenic acid (RA; C18:2c9,t11; also named c9,t11-conjugated linoleic acid (c9,t11-CLA) and vaccenic acid (VA; C18:1t11), and α-linolenic acid (ALA; C18:3n3), and to enhancing the n6 to n3 ratio, considering that a more balanced dietary intake of n6 relative to n3 is desired for optimal human health. Milk and dairy products also contain odd-chain fatty acids (OCFA), of which pentadecanoic (C15:0) and heptadecanoic (C17:0) acids have recently attracted scientists’ attention because of their potential activity against metabolic diseases in humans [4]. These OCFAs mainly originate from ruminal bacteria and are then distributed to tissues and consequently to ruminant-derived foods. For that reason, odd- and branched-chain fatty acids (OBCFA) are potential biomarkers of dairy fat intake [5] and biomarkers of ruminal fermentation [6].

Dairy products are also the major source of RA and VA in the human diet. The content of these FAs in sheep milk and dairy products, which is often higher than in cow and goat milk, sparks particular interest [7,8].

RA and VA are two of the main intermediate products arising from the biohydrogenation of polyunsaturated fatty acids (PUFA) by rumen bacteria [9]. In ruminants fed a typical forage diet, the major biohydrogenation intermediate present in ruminal contents is VA [10]. Part of the VA escaping from the rumen is converted to c9,t11-CLA in the mammary gland by the enzymatic reduction operated by the delta-9 desaturase. Investigations into animal and human cell lines reported several positive biological effects of c9,t11-CLA, especially RA [11,12]. Several beneficial effects of RA against enteropathy [13], atherosclerosis [14], cancer [15,16], and inflammation [17,18] have also been reported in animal and in vitro trials. Recently, positive effects of RA have also been observed in studies on humans, through the dietary inclusion of dairy products naturally rich in RA. In some cases, a negative correlation was observed between the consumption of dairy foods naturally enriched with c9,t11-CLA and the content of blood cholesterol [19,20] and inflammatory markers [17,21]. The beneficial properties of VA have also been reported [22,23,24]. Natural exposure of children to VA in early life reduces the risk of eczema [25]. Another important class of FA occurring in milk is PUFAs belonging to the ω-3 family, mainly represented by ALA. This FA has showed positive effects in terms of heart disease prevention [26,27,28] and metabolic disorder amelioration [29] in animal and human trials.

The diet is one of the most important factors affecting the quantity and quality of fat in sheep and goat milk [12,30,31]. Considering that the increase in beneficial FA in milk also depends on the dietary supply of PUFA, nutritional strategies including feed rich in these FAs are used with this aim in mind. In particular, good results have been obtained by using pasture-rich diets [31,32,33,34] or by dietary supplementation with plant lipid sources [30,31,35,36]. In addition, an interaction between alpha-s1-casein gene polymorphism and diet on goat milk FA composition has also been reported [37,38].

Considering the large impact of the feeding regimen on the milk FA composition and the recent advancements in this field, this bibliographic review provides: (i) a survey of the studies concerning feeding strategies that are useful for increasing the concentration of FA considered beneficial to humans (with particular attention to some PUFAs) in sheep and goat milk and dairy products; the work focuses in particular on the use of pasture, vegetable oil sources, and dietary inclusion of secondary metabolite-rich feeds; and (ii) a quantitative study concerning the main differences between the two species in terms of the feeding strategies that affect their milk’s FA profile.

## 2. Feeding Strategy to Improve the Fatty Acid Content in Sheep and Goat Milk

### 2.1. Effects of Grass and Forage on Milk Fat Quality

Evidence points to the positive impact of fresh pasture (grazed or provided as forage) on the milk concentration of VA and RA [31,32]; this is mainly due to the high amount of ALA in fresh grass and green forage. ALA is partially converted into VA by the biohydrogenation process occurring in the rumen. In the mammary gland, part of VA is converted to RA by the enzymatic reduction operated by the delta-9 desaturase. RA produced in this way represents the largest proportion of this FA in milk. The increase in VA and RA as a consequence of fresh grass consumption could also be related to the alteration of the rumen environment in other ways, as higher rumen pH occurs with increases in the forage to concentrate ratio [32].

**Figure 1:**
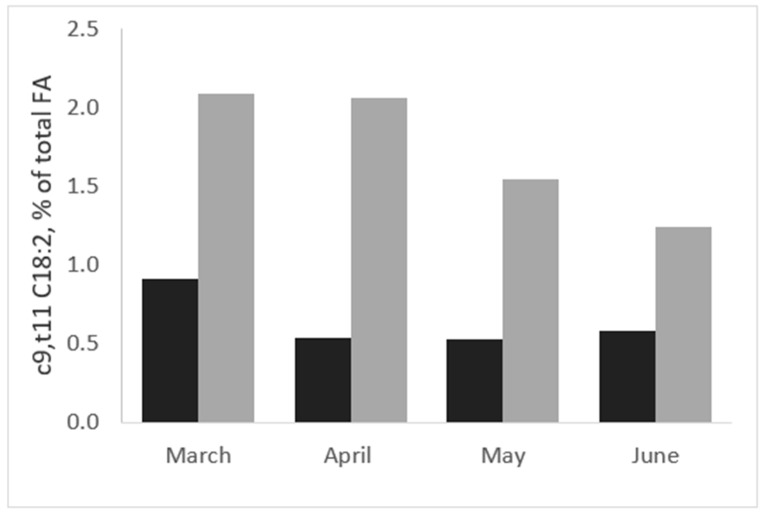
Rumenic acid (c9,t11-conjugated linoleic acid (c9,t11-CLA)) content (% of total fatty acids (FA)) in milk from sheep (dark grey) and goats (light grey) grazing the same pasture from March to June (adapted from Nudda et al. [40]).

The response of sheep to a pasture-based diet seems different to that of goats, considering that the RA content in sheep milk is normally higher than in goat and cow milk on pasture [39]. Sheep and goats maintained on the same mixed pasture composed of *Trifolium* sp. and *Lolium* sp. showed that the content of RA was 3.4-fold greater in ewes than in goats in April (2.15 vs. 0.64 g/100 of fat; *p* < 0.01), with lush pasture, whereas in May, when the amount and quality of pasture decreased, the differences in RA concentration were lower (0.84 vs. 0.79 g/100 of fat in sheep and goats, respectively [40]). Moreover, a different seasonal evolution of RA was observed in sheep milk [41] compared to goat milk [42], collected from different processing plants in Sardinia (Italy) from March to June (Figure 1); this pattern is similar to that reported from January to June for sheep and goats reared in semi-extensive systems in Greece [32].

**Figure 2:**
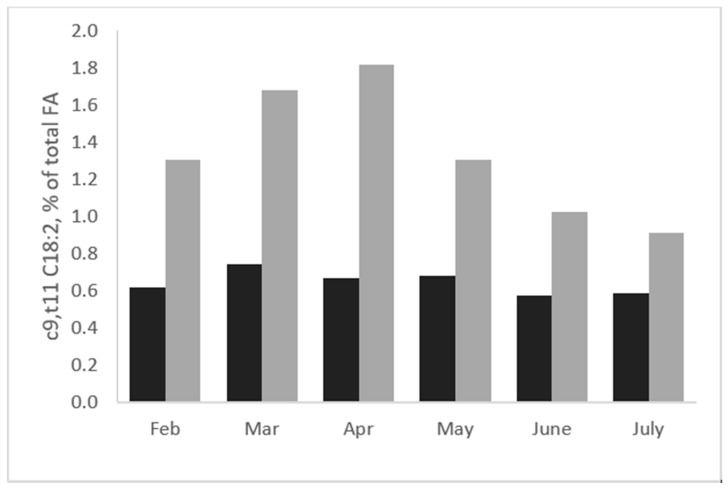
Evolution of rumenic acid (c9,t11-CLA, % of total FA) in milk of sheep (dark grey; adapted from Nudda et al. [41]) and goats (light grey; adapted from Nudda et al. [42]) from February to July.

The results observed in milk were confirmed by a survey carried out to compare the FA composition of sheep and goat cheese produced across the entire lactation period [33]. Indeed, the RA content in sheep cheese was higher compared to that of goat cheese during each month of production; the content of RA in sheep cheese reached the highest values in March and April, corresponding to the period when pasture availability and quality were the highest (Figure 2). On the other hand, the content of RA in goat cheese was nearly constant during all the months of production.

The differences in eating behavior during grazing, such as total intake, plant species selectivity, and meal timing, between sheep and goats could be an explanation for the different RA content found in the milk of the two species. Interestingly, the RA content increased as pasture intake increased, in both sheep [43] and goats [44], even if the response degree was greater in sheep than in goats. However, the increase in c9,t11-CLA content in milk is only partially explained by daily pasture intake, as ewes grazing on mixed or legume-based pasture had a higher content of PUFA in milk compared to ewes grazing on grass-based pasture [45]. This could be related to the higher content of PUFA in legumes than in grasses [46], with an evidently higher intake of PUFA with legume forage. It is established that goats on pasture have a markedly lower preference for legumes than grasses [47]; they showed a dislike for legume flavor and a preference for grass flavor in short-term cafeteria trials [48]. The aversion of goats to legumes, which are richer in the rumen precursors involved in c9,t11-CLA formation, could be one reason for the lower RA content in the milk of goats fed on legume-based pasture. Goats also select more woody species and prefer shrubland and less herbaceous plants compared to sheep [49]. Moreover, goats have smaller and more frequent meals than sheep [50], which allows for a more regular rumen pH pattern and a more complete biohydrogenation of unsaturated FA in the diet, reducing the RA content in their milk. However, the differences between the species must be studied using comparable conditions and the same feeding regime, as suggested by Szumacher-Strabel et al. [10]. Thus, the mechanisms explaining the differences between sheep and goats need further investigation and grounded experimental evidence to be corroborated.

### 2.2. Effects of Vegetable Oil on Milk Fat Quality

The inclusion of vegetable oils in the diet of dairy small ruminants could be considered a good strategy to increase energy in the diet and ameliorate the milk FA profile, especially when the content of unsaturated FA in the diet is low, which can occur when hay or silage is the main source of roughage in the diet. The quantity and physical form of the lipids included in the diet represent two of the main factors affecting the milk concentration of VA and RA, in both sheep and goats [30,51,52]; however as pointed by Cieslak et al. [53] the effect and the interaction with other dietary ingredients and supplements should be considered. Sunflower, linseed, safflower, soybean, and rapeseed grains and oils are the most widely used lipid supplements with the aim of increasing the milk concentration of VA, RA and, in general, of the unsaturated FAs in small ruminants [31,52,54]. Among these sources of PUFA, linseed, which is very rich in ALA, is the most used. By analyzing several publications (Table 1), the level of the increase in RA and VA using fat from linseed was similar in sheep and goats (+127% and +175% in sheep, and +167% and +262% in goats for RA and VA, respectively). The inclusion of linseed oil in both species is also effective at increasing ALA content and therefore reducing the n6 to n3 ratio (Nguyen et al., 2019) [55].

An interaction between milk protein polymorphism and the use of vegetable oils was evidenced. Numerous interactions, even if of limited quantitative extent, were evidenced between the alpha s1-casein genotype and extruded linseed fed to goats, and milk FA composition [60]. In addition, a breed effect on the milk FA profile and, consequently, of derived products, should not be neglected, as observed by Sinanoglou et al. [71] in sheep.

Table 2 summarizes the effects of the supplementation of sheep and goat diets with soybean or sunflower oil on the milk concentration of some FAs relevant for human health: VA, RA, LA, and ALA.

**Figure 3:**
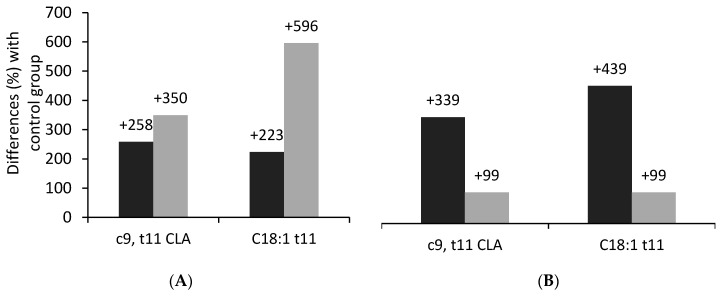
Average increment (% compared to control group) of rumenic acid (c9,t11-CLA) and vaccenic acid (C18:1t11) for soybean oil (**A**) and sunflower oil (**B**) supplementation in sheep (light grey) and goats (dark grey). Sheep data from [62,64,65,66,67,68,69]. Goat data from [30,59,72,73,74].

A comparison between the species shows that the dietary supplementation of sunflower and soybean oil determines the increase of VA and RA concentrations (Figure 3), even if the average increase in sheep milk was greater for soybean oil (+350% for RA, +596% for VA) than for sunflower oil, whereas, in goat milk, sunflower oil worked better (+339% for RA, +439% for VA). As expected, the n6 to n3 ratio has been increased by vegetable oils rich in LA (Table 2). However, the extent of the increase is markedly higher in goats than in sheep, as a result of the higher rumen escape of LA toward the mammary gland. Considering that soybean and sunflower oils have similar FA composition, the reasons for the observed differences among species remains unclear. Furthermore, in a recent in vitro experiment using rumen fluid, sunflower oil was more efficient than soybean oil at increasing the concentration of VA and RA [78]. As mentioned previously, sheep tend to have less frequent and greater meals than goats [50] and thus, possibly, less regular patterns of rumen pH and feed outflow.

Other lipid sources, such as safflower [79], pomegranate [57], hemp seed [80], and marine oils [54], are reported to be effective at increasing the c9,t11-CLA concentration in sheep milk, as reviewed by Nudda et al. [31] and Albenzio et al. [52]. Moreover, a combined supplementation of the diets with lipid sources and tannins could be another approach to increasing the content of unsaturated FA in sheep milk [81,82], because of their efficacy at modulating the biohydrogenation of PUFA.

### 2.3. Effects of Fish Oil and Algae Supplementation on the Fatty Acid Profile of Sheep and Goat Milk

The introduction of marine oils obtained from fish or algae to the diet of ruminants appears to be an attractive way to increase healthy FA in milk, making sheep and goat milk nutritionally healthier for the human diet compared to unsupplemented diets. Specifically, fish and algae oils are rich in ω-3 polyunsaturated fatty acids (ω-3 PUFA), a group of essential FA that cannot be synthesized de novo by humans and, therefore, need to be acquired from the diet [83]. Several studies evidenced the role of ω-3 PUFA in the prevention of heart disease and metabolic disorder, in the inhibition of inflammatory process, and in the reduced risk of developing Alzheimer’s disease, as widely reviewed by Nguyen et al. [55]. Considering the low consumption of seafood in the human diet, the natural enrichment of other common human foods in ω-3 PUFA from marine-derived supplements can be considered an important opportunity to increase the amount of these essential fatty acids in the human diet, as suggested by Nguyen et al. [55]. In particular, being rich in ω-3 PUFA, fish and algae oils promote the outflow of FA of nutritional interest, such as eicosapentaenoic acid (EPA; C20:5n3) and docosahexaenoic acid (DHA; C22:6n3), from the rumen to the blood and then to the mammary gland. Thus, the incorporation of these beneficial FAs in milk appears to be a promising way to enhance the quality of foods of animal origin and provides an excellent opportunity to increase the level of ω-3 in the diet of non-breastfed infants [84].

The amount of these long-chain FAs in milk fat seems to be enhanced as a consequence of the inclusion of rumen-protected marine oil, rich in EPA and DHA, compared to milk from untreated sheep and goats (Table 3). In goats, the use of rumen-protected fish oil from tuna was able to transfer EPA and DHA at a rate ranging from 3.5% to 7.6%, for EPA and DHA, respectively [84]. Recently, other studies observed that the inclusion of marine algae powder [85] or fish oil [86] in the diet of early lactating goats promoted the passage of EPA and DHA into the milk. In dairy goats, the supplementation of *Schizochytrium limacinum* marine algae (15 g/head/day) increased the content of DHA in milk and positively influenced the n6 to n3 ratio in goats kept indoors or on pasture compared to unsupplemented diets [87]. Similarly, in ewes, rumen-protected tuna oil supplementation increased the level of EPA and DHA in milk until 24 h after the postprandial period and reached a peak 10 days afterward (4.7 ± 0.5 g/kg and 19 ± 0.9 g/kg, respectively), whereas no EPA and DHA was detected in milk from untreated sheep [88]. In the same experiment, six days after feeding stopped, the concentration of EPA and DHA in milk was still significantly enriched (2.3 and 6.5 g/kg, respectively), in contrast to linoleic acid (LA, C18:2n6) and ALA, which returned to their preprandial content. As pointed out by Nguyen et al. [55], supplementation with fish oil is more profitable than the use of marine algae. However, it is very difficult to increase EPA and DHA in milk because of the extent of ruminal biohydrogenation [89]. Although it is poor in c9,t11-CLA precursors, fish oil is able to increase milk RA (Table 3), probably because it inhibits rumen bacteria activity, thus lowering the biohydrogenation of VA to SA, and favors the desaturation of VA to RA at the mammary gland level. In fact, the inclusion of fish oil in the diet of lactating Saanen [86] and Alpine crossbreed goats [73] increased the content of RA and VA. The supplementation of *Chlorella kessleri* micro-alga (10 g/kg of DMI) positively affected the concentration of stearic acid (C18:0) and VA in the milk of Hungarian Native goats [90].

Similarly, the inclusion of three incremental doses of marine algae (8, 16, and 24 g/kg DM) with 25 g/kg DM of sunflower oil in the diet of mid-lactating Assaf ewes increased the concentration of VA and RA in their milk [91]. As can be observed in Table 3, the n6 to n3 ratio is generally lowered in both species, due to the increase of the ω-3 PUFA, in particular EPA and DHA. However, the inclusion of marine oil in the diet of ruminants also induces milk fat depression, as observed in lactating ewes [92] and goats [93], discouraging its use under practical conditions [92].

**Table 3:** Effect on rumenic, alpha-linolenic, and long-chain fatty acid milk concentration in sheep and goats fed a marine lipid sources supplementation. Data are reported as the proportional difference between the supplemented group, at the respective dose, and the non-supplemented control group, with dose 0.

Species	Diet ^2^	Milk Fatty Acids (%) ^1^				References
		RA	ALA	EPA + DHA	n6:n3	
Goats	55.5 g/day SO + 11.1 g/day FO	609	13	260	61	[73]
	27.0 g/day *Chlorella kessleri* microalga	28	33	120	−18	[90]
	10.0 g/day *Chlorella vulgaris* microalgae	−11	−6	7	18	[94]
	10.0 g/day *Japonochytrium* sp. microalgae	−1	−12	21	−11	[94]
	50.0 g/day FO		−9		−71	[93]
	20.0 g/day FO	1377			−74	[86]
	40.0 g/day MA		18		−71	[85]
Sheep	30.0 g/day FO	146	18	500	−21	[95]
	45.0 g/day FO	322	8	960	−15	[95]
	6.0 g/day DHA			2863	−60	[88]
	25.0 g SUN + 8.0 g MA	126	−10	200	9	[91]
	25.0 g SUN + 16.0 g MA	110	−12	556	−30	[91]
	25.0 g SUN + 24.0 g MA	162	−17	700	−31	[91]
	0.25% FO + 3.75% SO	10	0	400		[96]
	0.50% FO + 3.50% SO	22	0	500		[96]
	0.75% FO + 3.25% SO	7	−8	600		[96]
	25.0 g SO + 8.0 g MA/kg DM	65	−2	389	−23	[97]

^1^ RA = rumenic acid (c9,t11-CLA); ALA = α-linolenic acid (C18:3n3); EPA = eicosapentaenoic acid (C20:5 n3); DHA = docosahexaenoic acid (C22:6 n3). ^2^ SO = soybean oil; FO = fish oil; MA = marine algae; SUN = sunflower oil.

### 2.4. Effects of Essential Oils on the Fatty Acid Profile of Sheep and Goat Milk

Another promising category of feed additives that can positively affect the milk fatty acid concentration in both sheep and goats is the essential oils (EO). These bioactive compounds are volatile aromatic substances produced by plants and can be divided into two main classes: terpenoids (most abundant) and phenylpropanoids, which are present in flowers, leaves, pulp, bark, fruits, roots, seeds, etc. [98].

The inclusion of EO in lactating ruminant diets has been tested mostly in view of their antimicrobial and antioxidant activity [99]. Recently, their inclusion in the ruminant diets has resulted from a focus on the improvement of rumen fermentation and protein metabolism and on the increase in conjugated linoleic acid in milk and meat [98]. Despite their potential, the inclusion of EO in ewes and goats diet is still not widespread due to variability in terms of the parts of the plants utilized, EO extraction method, purity, dose of EO used, chemical stability, etc., as widely reviewed by [99].

Especially in ewes, there is a lack of knowledge about the effects of the use of EO, as feed supplements, on milk production and composition (Table 4). Boaventura Neto [100], reviewing the literature on the use of essential oils in small and large ruminants, observed that small ruminants responded better than cows to essential oil supplementation, probably due to the fact that small ruminants have, proportional to their body size, faster rumen passage than large ruminants, which probably increased their digestion at the intestinal level. In addition, the same author observed that in all studies conducted on sheep and goats, which were the object of the literature review, the addition of EO in the diet reduced the biohydrogenation of FA, the opposite to what was observed in dairy cows.

The inclusion of leaves of three aromatic plants (*Melissa officinalis* L., *Ocimum basilicum* L., and *Thymus vulgaris* L.) as a source of EO at three increasing levels (50, 125, and 200 g/day, DM basis), in the diet of lactating Sarda dairy sheep was effective at increasing the milk concentration of BCFA, ω-3 PUFA, and the sum of CLA isomers [101]. In another study, the inclusion of orange-peel EO in the diet of lactating Chios ewes decreased the content of unsaturated FA and improved the antioxidant status in blood and milk [102].

**Table 4:** Effects of essential oils on the fatty acid profile of goat and sheep milk. Data are reported as the proportional difference between the supplemented group, at the respective dose, and the non-supplemented control group, with dose 0.

Species	Source	Dietary EO ^2^	Milk Fatty Acids (%) ^1^								References
			SFA	MUFA	PUFA	SA	VA	RA	LA	ALA	
Goats	*Rosmarinus officinalis* spp.	10% of control diet	−2	1	38	−4			36	0	[103]
	*Rosmarinus officinalis* spp.	20% of control diet	−2	−1	54	4			55	23	[103]
	Cuminum cyminum	1.27 g/kg DMI	−2	2	22	−4	7	25	23	23	[104]
	Cuminum cyminum	2.53 g/kg DMI	−3	1	25	−7	7	24	25	29	[104]
	Garlic oil	0.57 g/kg DM	0	1	4	0	1	7	−1	10	[105]
	Garlic oil	1.14 g/kg DM	0	0	3	1	3	12	−1	5	[105]
	Garlic oil	1.71 g/kg DM	−1	1	6	2	4	12	−1	10	[105]
	Citral oil	0.08 mL of EO/kg BW				3	−16	−15	−3		[106]
	Citral oil	0.16 mL of EO/kg BW				2	−3	−4	−3		[106]
Sheep	Citral oil	0.24 mL of EO/kg BW				3	−9	−4	−5		[106]
	α-pinene, limonene, β-caryophyllene	1 mL of each in a 10-mL soya bean oil mixture	0	6	5	−3			−8	37	[107]
	Carum seeds	High dose	−7	38	5	71	86	10	6	−12	[100]
	Coriandrum seeds	High dose	−7	45	−15	97	152	−17	−16	−21	[100]
	Satureja leaves	High dose	0	0	−5	4	−9	−15	−13	0	[100]
	Orange peel	150 mg/kg	5	−11	−10	−13	3	1	−12	−17	[102]
	Orange peel	300 mg/kg	5	−12	−15	−12	−10	−10	−15	−17	[102]
	Orange peel	450 mg/kg	1	−3	−7	−1	−8	−3	−8	−9	[102]

^1^ SFA = saturated fatty acids; MUFA = monounsaturated fatty acids; PUFA = polyunsaturated fatty acids; SA = stearic acid (C18:0); VA = vaccenic acid (C18:1t11); RA = rumenic acid (c9,t11-CLA); LA = linoleic acid (C18:2n6); ALA = α-linolenic acid (C18:3n3). ^2^ EO = essential oils; DMI = dry matter intake; BW = body weight.

The literature data concerning the effect of EO on goat milk’s fatty acid profile are relatively more consistent (Table 4). Except for one study that observed no effect of citral oil on the milk FA profile in Saanen goats [106], the inclusion of EO in the diet improved the milk fatty acid profile in goats. For example, the inclusion of a methanolic extract of cumin seeds at two dosages (12.7 and 25.3 g/kg DMI) reduced the concentration of saturated FA (SFA) and increased that of PUFA, monounsaturated FA (MUFA), and the PUFA: SFA ratio in goat milk [104]. The inclusion of a mixture of three of the most widespread terpenes in forage (α-pinene, limonene and β-caryophyllene) in the diet of lactating goats increased C18:3, c9,t11-CLA, and MUFA concentrations in their milk [107]. Other studies reported that the inclusion of garlic oil (0.57, 1.14, or 1.71 g/kg DM) in the diet of early lactating goats increased C18, c9,t11-CLA, t10,c12-CLA, MUFA, and PUFA in milk as garlic oil increased [105]. Finally, 10% *Rosmarinus officinalis* L. leaf supplementation increased the C18:2 and PUFA content in the milk of goats, while the same supplementation at a dose of 20% also increased C17:0 [103]. The composition of milk FA in lactating goats was positively modified after the inclusion of ginger, cinnamon, and garlic oils [108]. However, the fatty acid profile of treatments shown in this work is not consistent with the typical fatty acid profile of ruminant milk (i.e., 0% of CLA, and more than 21% of trans-oleic acid (C18:2 n9t) in the control diet).

The n6 to n3 ratio, on average, increased in the milk of goats (+6%), whereas it did not change in sheep milk (−1%) after supplementation with essential oil. However, these data should be carefully interpreted, since positive or negative effects on the n6:n3 ratio can also be observed within species.

In summary, very few studies investigating possible differences have focused on the effects of EO on sheep, whereas relatively more has been published about goats. On the basis of this evidence, the addition of EO sources (as oil or plants) to goats’ diet seems to markedly affect the milk FA profile, especially increasing MUFA, PUFA, VA, RA, and linoleic acid (LA; C18:2n6), whereas the addition of aromatic plants to sheep’s diet affected the milk FA profile to a less significant extent.

### 2.5. Effects of Dietary Polyphenols on the Fatty Acid Profile of Sheep and Goat Milk

Another factor that can affect milk composition in ruminants is the inclusion of polyphenols in their diet. As products of the plant secondary metabolism, these compounds can naturally occur in the diet of herbivorous, and particularly grazing, animals. Polyphenolic compounds can range from simple phenolics (e.g., ellagic and gallic acids) to dimeric or oligomeric compounds (e.g., procyanidins and lignans), or polymeric compounds characterized by a high molecular weight [109]. The occurrence and effects of these compounds in the diet of ruminants have been widely reviewed, with a particular focus on the tannins [110,111]. The first studies on the polyphenols’ effects on animal diet refer to dose-dependent antinutritional effects: the reduction of protein and carbohydrate digestion, negative impact on feed intake, and milk production. These effects are the consequence of the low feed palatability caused by the astringency arising from the bonds between the tannins and salivary proteins [112]. Different effects of polyphenols on DM intake between sheep and goats have been reviewed, referring to the major negative impact on sheep [113]; this effect is possibly associated with sheep not being well adapted to digesting tannin-rich plants [114]. However, independently of the ruminant species, more recent research has showed the beneficial effects of moderate levels of dietary polyphenols on ruminant health [115], performance [111], nitrogen utilization [116], and the quality of derived products, in particular on the lipid fraction [93]. These compounds act by modulating the ruminal lipid metabolism, and, consequently, the FA composition of milk. Dietary polyphenols can reduce or inhibit the activity and growth of rumen microorganisms, which are known to play an important role in the biohydrogenation of PUFA [117,118,119]. Although several studies have been carried out on this topic, the effectiveness of plant secondary compounds in improving milk lipid quality is still controversial. This is likely the consequence of the diversity existing among the ruminant species considered in the studies and the wide variety in active compounds, dosage, basal diet composition, and duration of the experiments [91]. A clarifying example of the complex interactions among diverse factors is shown by the analysis of three studies on the effects of dietary tannins on the milk FA profile in sheep. Equal doses of chestnut tannins fed to dairy sheep had different effects on RA, causing no difference or increasing it [81,82,120], possibly due to differences in breeds (e.g., Comisana and Sarda), basal diets (hay vs. pasture) and type of oil supplementation (extruded linseed vs. soybean oil). The effects of dietary polyphenols on the FA composition of sheep and goat milk are summarized in Table 5. Differences between the two species are not easily seen. The main reason is that the existing studies were carried out under very different experimental conditions, in particular different sources of polyphenolic compounds; no work exists, to date, comparing the two species under identical conditions.

In general, polyphenols have positive effects on the PUFA concentration in both sheep and goat milk. In particular, the increase in LA and ALA concentrations, calculated as the proportional difference between the treatment and the control group, were, on average, +7% and +33%, in sheep and +17% and +24% in goats, respectively. It should be noted that, although the average increase of LA in goats appears higher than that of sheep, such differences are not statistically significant, except in the work of Alipanahi et al. [126]. The increase in the PUFA concentration in milk is in accordance with the mechanism proposed by some authors about the effect of polyphenols on the biohydrogenation of unsaturated FA. The initial reduction of PUFA biohydrogenation observed in vivo [131], related to the effect of dietary polyphenols on the ruminal bacteria responsible for the reactions, inhibited the last step of ruminal biohydrogenation of PUFA, i.e., the enzymatic reaction that reduces VA to SA, by polyphenols (condensed tannins, in particular). The two PUFAs, LA and ALA, derive from the diet, and an inhibitory effect on ruminal biohydrogenation leads to their increase in milk. Furthermore, in both species, a general trend of an increase in MUFA and reduction in SFA can be observed.

The generally higher increase of LNA compared to LA reduced the n6:n3 ratio in both sheep and goats (means of −8% and −12%, respectively).

**Figure 4:**
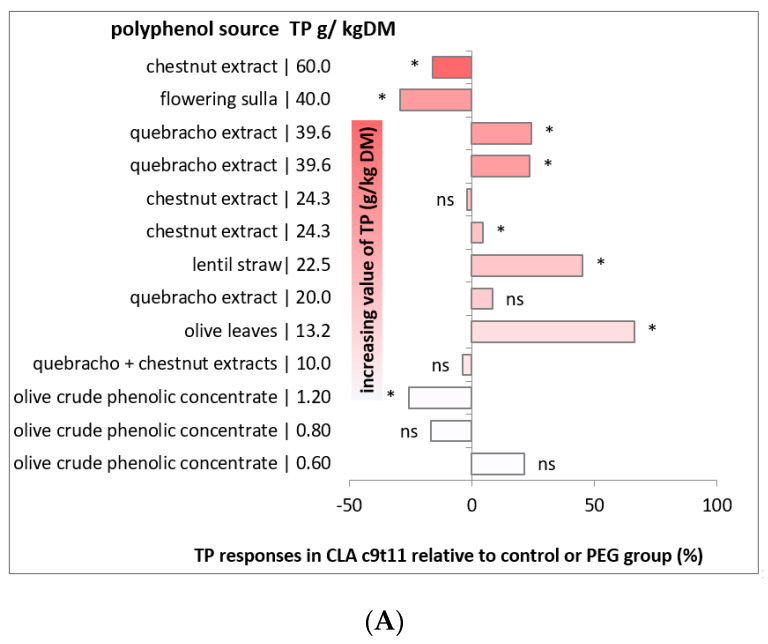

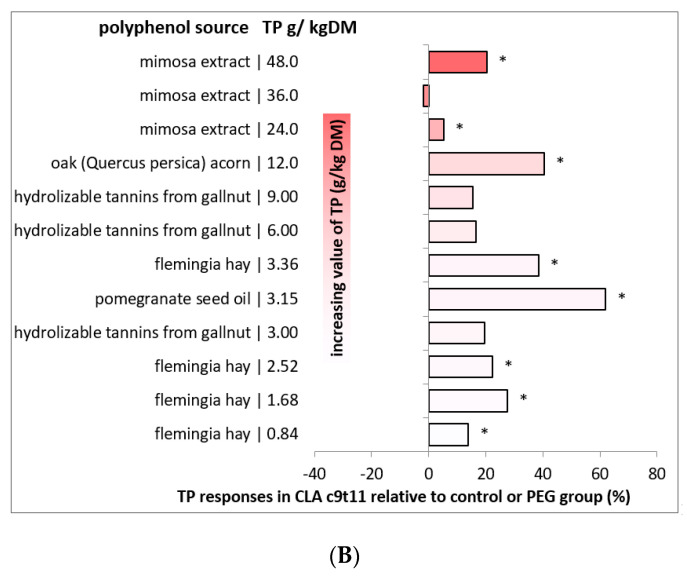
Effect of total polyphenol (TP) concentration on sheep (**A**) and goat (**B**) milk concentration of rumenic acid (RA; c9,t11-CLA). Data are reported as the proportional difference between the supplemented group, at the respective dose, and the non-supplemented control group or a group supplemented with polyethylene glycol (PEG); * indicates a significant difference (*p* < 0.05) compared with the control group, ns indicates no significant variations (*p* > 0.05). (**A**) Mix: quebracho and chestnut extract [122]; olive leaves [121]; quebracho extract [82,120,123]; lentil straw [121]; chestnut extract [82,120]; flowering sulla [118]. (**B**) Flemingia hay [128]; gallnut [125]; pomegranate seed oil [57,129]; oak [126]; mimosa extract [127].

The general reduction of SFA, even if of a low extent (mean of −1% and −3% in sheep and goats, respectively), could be related to the decrease in the SA content, due to the inhibition of the extent of enzymatic reduction of VA, but also to a possible depressant effect played by PUFAs and their biohydrogenation intermediates on fat synthesis in the mammary gland [132,133]. In fact, all short and medium saturated FAs (from C6 to C14) and almost 50% of C16:0 are synthetized de novo in the mammary gland [134]. In both species, the pattern of FA seems to be in agreement to these pathways, even if in goats the first seems to be less important—considering that, in the reported research, there is no evident reduction in SA concentration, and in some cases it increases [119,122]. The reason could be the possible biohydrogenation of other C18:1 isomers besides VA. Another complication to the understanding of the causes of the FA modification is related to the enzymatic activity of the mammary gland; in fact, the concentration of SA, VA, and RA in milk depends not only on rumen biohydrogenation processes, but also on the activity of the mammary enzyme stearoyl-CoA 9-desaturase, whose major substrates are SA and VA and final products are oleic acid (OA; C18:1c9) and RA, respectively. The activity and expression of stearoyl-CoA 9-desaturase is regulated by different factors, including the concentration of dietary PUFAs [135], which can differ among treatments or experiments, but also, indirectly, by the dietary tannins via modulation of the absorbed fatty acids and protein level [136]. Moreover, different activity of stearoyl-CoA 9-desaturase has been reported between sheep and goats, with sheep exhibiting a higher milk concentration of RA than goats, correlating to the higher levels of stearoyl-CoA desaturase mRNA observed in sheep than in goats under the same dietary treatment [137]. Figure 4 reports the variation in percentage (negative or positive) of the RA content in the milk of the two species in response to the different concentrations of dietary polyphenols reported in different studies. With a few exceptions, the inclusion of polyphenols in the diet of sheep and goats had positive effects on the RA concentration, with values that increased from 5% to 66%. A significant decrease in the RA concentration was observed in the milk of sheep fed a high level of hydrolysable tannins from chestnut extract [81] or condensed tannins from flowering Sulla [118]; this could be because dietary polyphenols reduced the PUFA biohydrogenation in the first step of the process, as suggested by the increase in the milk concentration of PUFAs and the decrease in PUFA intermediates, including RA and VA [81]. Similar results were also obtained by [124] in sheep fed very low levels of olive crude phenolic concentrate (1.2 g/kg DM). In Figure 4, it can be seen that increasing values of dietary polyphenol concentrations do not match a linear increase in FA content in milk. These last observations and considerations emphasize the complex pattern of the effects of polyphenols on the milk FA composition. Different compounds could exert similar effects at very different dosages; on the other hand, different dosages of the same compounds may have a different effect on the FA composition, also acting by different inhibition ways of biohydrogenation. The substantial diversity of the results, and the generally positive effects of moderate levels of polyphenols in the diet, suggest that these compounds are useful for improving the quality of milk FA composition in sheep and goats, without evident differences between sheep and goats. Moreover, to achieve the best results, preliminary experiments should be carried out to assess for each polyphenol source the best method of application, such as the dosage of the supplementation and the composition of the basal diet.

Polyphenols are secondary plant metabolites that can confer oxidative stability to dairy products [138]. Indeed, the nutritional value and functional properties of milk can change due to deterioration and oxidation processes that occur during production or storage. Oxidation of milk fatty components represents the cause of the development of rancidity, off flavor, and toxic compounds. This is of particular concern for milk with high PUFA content, due to the susceptibility of double bonds to peroxidation. The effects of polyphenolic extracts or feeds containing polyphenolic compounds (e.g., fresh herbage) on the oxidative stability of milk have been investigated recently. Sulla fresh forage diet improved the total antioxidant capacities of goat milk [139]. A reduction in the extent of lipid oxidation was observed in milk from sheep fed grape seed residues [140], confirming previous observations in dairy cows supplemented with grape residue [141]. The inclusion of hemp seed and hemp cake in the sheep diet also improved antioxidant activity in milk [80].

## 3. Conclusions

Notwithstanding the fact that sheep and goats are both small ruminants, the strategies to design a desirable fatty acid composition of milk fat by nutritional means could be quite different in these two animals. Sheep seem to have higher improvements of RA and VA after grazing fresh herbage compared to goats, probably because of their diverse grazing habit: sheep prefer legumes, richer in PUFAs compared to grass, which is preferred by goats. Not only the grazing habit but also differences in the FA metabolism and fermentation parameters between sheep and goats can influence the FA composition. On the other hand, goats and sheep responded positively to both soybean and sunflower oil supplementation, with higher improvements in RA and VA milk content in sheep for soybean oil and in goats for sunflower oil. Supplementation with linseeds and linseed oil represents a valuable strategy for a concomitant improvement in milk CLA and the n6:n3 ratio in both species. Marine oil supplements dramatically increased the EPA + DHA concentration, making them a potentially valuable resource for modulating the content of these very important FAs in human nutrition. However, they induced milk fat depression, suggesting their utilization in rumen-protected forms. The literature is quite rich in experimental data dealing with the effect of essential oils on the FA milk profile of goats rather than sheep; in general, these substances seem to boost the MUFA and PUFA milk fat concentrations. Polyphenols have also been used to improve the nutritional FA profile of milk, especially the c9,t11-CLA content, in both goats and sheep; however, there is not a dose-related response due to both the huge variability of chemical forms of the compounds included in this class and the complex interferences among polyphenol sources, diet, and the rumen microbiome. Polyphenols can also be useful to reduce the extent of lipid oxidation of milk fat, a serious problem when milk fat unsaturation increases due to the reduced activity of the rumen biohydrogenation process.

## Figures and Tables

**Table 1:** Effect on rumenic, vaccenic, linoleic, and alpha linolenic acid milk concentrations in sheep and goats fed a linseed supplementation. Data are reported as the proportional difference between the supplemented group, at the respective dose, and the non-supplemented control group, with dose 0.

Species	Dose	Form ^2^	Fat/d	Milk Fatty Acids (%) ^1^					References
				RA	VA	LA	ALA	n6/n3	
**Goats**	100 g/d	EL	17 g/d	52	76	3	78	−42	[35]
	200 g/d	EL	34 g/d	67	99	3	120	−53	[35]
	160 g/d	EL	56 g/d	112	148	13	132	−86	[56]
	200 g/d	EL + P	70 g/d	55	100	1	145	−59	[36]
	47 g/d	Oil	47 g/d	100	26	12	98	−44	[57]
	360 + 40 g/d	EL	126 g/d	103	187	5	288	−73	[58]
	3.4% DMI ^3^	Oil	102 g/d	133	190	0	325	−76	[30]
	130 g/d ^4^	Oil	130 g/d	300	439	−38	11	−44	[59]
	130 g/d ^5^	Oil	130 g/d	213	358	−21	263	−78	[59]
	511 g/d	EL	197 g/d	690	1268	−9	415	−82	[60]
	30 g/d ^6^	Oil	30 g/d	48	75	−2	89	−48	[61]
	30 g/d ^7^	Oil	30 g/d	135	183	−13	102	−57	[61]
**Sheep**	210 g/d	EL	79 g/d	50	67	52	100	−31	[62]
	200 g/d	EL	70 g/d	213	294	11	153	−56	[63]
	100 g/d	EL + P	–	28	42	245	48	134	[64]
	63−70 g/d	Oil	63 g/d	308	447	−12	106	−57	[65]
	29 g/d	EL	11 g/d	9	30	0	14	−13	[66]
	58 g/d	EL	22 g/d	18	65	9	21	−10	[66]
	84 g/d	EL	32 g/d	73	83	9	43	−24	[66]
	210 g/d	EL	74 g/d	247	273	0	168	−63	[67]
	55 g/d	Oil	55 g/d	239	251	−6	103	−54	[68]
	128 g/d	Oil	45 g/d	141	256	−25	202	−75	[69]
	200 g/d	EL	70 g/d	74.84	122	−3	43	−33	[70]

^1^ RA = rumenic acid (c9,t11-CLA); VA = vaccenic acid (C18:1 t11); LA = linoleic acid (C18:2n6); ALA = α-linolenic acid (C18:3n3). ^2^ EL = extruded linseed; *p* = pasture. ^3^ DMI = dry matter intake. ^4^ control and treatment diets based on natural grassland hay. ^5^ control and treatment diets based on maize silage. ^6^ control and treatment diets characterized by high starch concentrate. ^7^ control and treatment diets characterized by high neutral detergent fiber (NDF) concentrate.

**Table 2:** Effect on rumenic, vaccenic, linoleic, and alpha linolenic acids’ milk concentrations in sheep and goats fed a soybean or sunflower supplementation. Data are reported as the proportional difference between the supplemented group, at the respective dose, and the non-supplemented control group, with dose 0.

Species	Dose	Form ^2^	Milk Fatty Acids (%) ^1^					References
			RA	VA	LA	ALA	n6/n3	
Goats	60 g/d	SO	199	214	14	−10	30	[72]
	55 + 11 g/d	SO + FO	609	466	34	13	15	[73]
		SW	−33	−10	50	0	24	[30]
		SUN‒oil	283	290	55	25	9	[30]
	130 g/d	SUN‒oil	351	472	23	−15	934	[74]
	130 g/d	SUN‒oil	384	554	16	−41	226	[59]
Sheep	100 g/d	SO	316	803	15	−38		[75]
	63‒70 g/d	SO	562	736	59	2	54	[65]
	60‒165 g/d	SO	171	242	13	−23	294	[51]
	182 g/d	SUN—seeds	130	67	78	56	25	[62]
	65 g/d	SUN—oil	138	195	6	−12	21	[76]
	28.8 g/d	SUN—oil	29	36	12	31	−14	[77]

^1^ RA = rumenic acid (c9,t11-CLA); VA = vaccenic acid (C18:1t11); LA = linoleic acid (C18:2n6); ALA = α-linolenic acid (C18:3n3). ^2^ SO = soybean oil; FO = fish oil; SW = whole soybean; SUN = sunflower.

**Table 5:** Effect of dietary total polyphenols on fatty acid profile of sheep milk. Data are reported as the proportional difference between the supplemented group, at the respective dose, and the non-supplemented control group, with dose 0.

Specie	Polyphenol Source	Dietary TP ^2^	Milk Fatty Acids (%) ^1^								References
			SFA	MUFA	PUFA	SA	VA	RA	LA	ALA	
Sheep	Flowering sulla	40.0		−4	3	11	−29	−29	18	45	[118]
	Lentil straw	13.2	−3	11	12	−7	25	45	9	79	[121]
	Olive leaves	22.5	−1	−1	16	−35	48	66	6	197	[121]
	Quebracho and Chestnut extracts	10.0	−1	−3	−1	4	2	−4	4	6	[122]
	Quebracho extract	20.0	−2	7	3	3	12	8	2	2	[123]
	Quebracho extract	39.6	−3	4	15	−11	14	24	9	16	[120]
	Chestnut extract	24.3	0	0	1	9	7	−2	3	3	[120]
	Quebracho extract	39.6	−3	4	15	−11	14	24	9	16	[82]
	Chestnut extract	24.3	0	0	1	9	7	−2	3	3	[82]
	Olive crude phenolic concentrate	0.6				−6	26	21	7	−4	[124]
	Olive crude phenolic concentrate	0.8				0	−19	−17	16	16	[124]
	Olive crude phenolic concentrate	1.2				9	−22	−26	18	24	[124]
Goat	Gallnut	3.0	−4	5	5	−2	24	24	−5		[125]
	Gallnut	6.0	−4	6	15	11	25	20	12		[125]
	Gallnut	9.0	−5	8	19	7	22	18	18		[125]
	Oak acorn	12.0				−25	48	68	13	22	[126]
	Mimosa extract	24.0	−1	1	11	11	20	5		13	[127]
	Mimosa extract	36.0	−2	3	24	16	20	−2		35	[127]
	Mimosa extract	48.0	−5	8	38	15	30	7		48	[127]
	Flemingia hay	0.8				4	14	16	9	16	[128]
	Flemingia hay	1.7				8	31	38	23	33	[128]
	Flemingia hay	2.5				−5	23	29	9	14	[128]
	Flemingia hay	3.4				14	77	62	20	32	[128]
	Pomegranate seed oil	3.2	−5	7	39	1	37	131	8	110	[57,129]
	Pomegranate seed pulp	4.7	−1	4	31	13	121	562			[130]
	Pistachio hulls	26.6	1	10	−1	−1	104	431			[130]
	Tomato pomace	3.2	−5	24	−3	14	100	400			[130]

^1^ SFA = saturated fatty acids; MUFA = monounsaturated fatty acids; PUFA = polyunsaturated fatty acids; SA = stearic acid (C18:0); VA = vaccenic acid (C18:1t11); RA = rumenic acid (c9,t11-CLA); LA = linoleic acid (C18:2n6); ALA = α-linolenic acid (C18:3n3). ^2^ TP = total polyphenols (g/kg DM).
